# Diagnostic performance of ultrasonography, dual-phase ^99m^Tc-MIBI scintigraphy, early and delayed ^99m^Tc-MIBI SPECT/CT in preoperative parathyroid gland localization in secondary hyperparathyroidism

**DOI:** 10.1186/s12880-020-00490-3

**Published:** 2020-08-03

**Authors:** Rongqin Zhang, Zhanwen Zhang, Pinbo Huang, Zhi Li, Rui Hu, Jie Zhang, Wanglin Qiu, Ping Hu

**Affiliations:** 1 Department of Nuclear Medicine, The Sixth Affiliated Hospital, Sun Yat-sen University, Guangzhou, 510655 China; 2 Department of Hepatobiliary Surgery, Sun Yat-sen Memorial Hospital, Sun Yat-sen University, Guangzhou, 510120 China

**Keywords:** Secondary hyperparathyroidism, Ultrasonography, Dual-phase ^99m^Tc-MIBI scintigraphy, ^99m^Tc-MIBI SPECT/CT

## Abstract

**Background:**

Secondary hyperparathyroidism (SHPT) usually requires parathyroidectomy when drug regimens fail. However, obtaining an exact preoperative map of the locations of the parathyroid glands is a challenge. The purpose of this study was to compare the diagnostic performance of US, dual-phase ^99m^Tc-MIBI scintigraphy, early and delayed ^99m^Tc-MIBI SPECT/CT in patients with SHPT.

**Methods:**

Sixty patients with SHPT who were undergoing dialysis were evaluated preoperatively by US, dual-phase ^99m^Tc-MIBI scintigraphy, early and delayed ^99m^Tc-MIBI SPECT/CT. Postoperative pathology served as the gold standard. The sensitivity, specificity, and accuracy were determined for each method. Spearman correlation analysis was used to analyse the correlation of hyperplastic parathyroid calcification with serum alkaline phosphatase (ALP) and parathyroid hormone (PTH).

**Results:**

A total of 229 lesions in 60 patients were pathologically confirmed to be parathyroid hyperplasia, with 209 lesions in typical sites, 15 lesions in the upper mediastinum and 5 lesions in the thyroid. A total of 88.33% (53/60) of patients had four lesions. US, early and delayed ^99m^Tc-MIBI SPECT/CT had significantly higher sensitivity and accuracy than dual-phase ^99m^Tc-MIBI scintigraphy (*P* < 0.001). Furthermore, early ^99m^Tc-MIBI SPECT/CT had significantly higher sensitivity (*P* < 0.001) and accuracy (*P* = 0.001 and *P* < 0.001) than US and delayed ^99m^Tc-MIBI SPECT/CT. In patients with ectopic hyperplastic parathyroid glands, the sensitivity of early ^99m^Tc-MIBI SPECT/CT (90%) was significantly higher than that of US (55%) and dual-phase ^99m^Tc-MIBI scintigraphy (50%) (*P* < 0.05). The Spearman correlation results showed a significant albeit weak association between calcification and both serum PTH and ALP (*P =* 0.002).

**Conclusion:**

The ability of early ^99m^Tc-MIBI SPECT/CT to detect hyperplastic parathyroid glands in patients with SHPT is superior to that of US, dual-phase ^99m^Tc-MIBI scintigraphy and delayed ^99m^Tc-MIBI SPECT/CT; furthermore, dual-phase ^99m^Tc-MIBI SPECT/CT is not essential.

## Background

Secondary hyperparathyroidism (SHPT) is a complex disease due to increased parathyroid hormone (PTH) production, which affects the metabolism of calcium and phosphorus and causes further abnormal PTH secretion, usually leading to 4-gland hyperplasia [1]. Severe SHPT mainly occurs in chronic kidney disease (CKD), which has been reported as a key causal factor of bone disease, muscle weakness, neurologic dysfunction, and soft tissue and vascular calcification, which increase cardiovascular morbidity and mortality [2, 3]. The National Kidney Foundation Kidney Disease Outcomes Quality Initiative (KDOQI) suggested parathyroidectomy to treat severe SHPT when drug regimens fail [4], and total parathyroidectomy has shown a minimum risk of postoperative relapse (0–4%) [5]. However, parathyroidectomy for SHPT is less satisfactory, and the occurrence of persistent SHPT after parathyroidectomy has been reported to be 0.4–25% [6]. Surgical failure is mainly due to the difficulty of resecting all parathyroid glands, especially because of the existence of supernumerary (more than 4) and ectopic parathyroid glands. The incidence of ectopic and supernumerary glands in patients with end-stage renal disease has been reported to be 17.5–39.3% and 6.3–37%, respectively [7–9]. Therefore, preoperative imaging and accurate localization are critical to a successful operation.

Parathyroid glands can be detected with multiple modalities, such as ultrasonography (US), computed tomography (CT) and magnetic resonance imaging (MRI), but the performance of these anatomical examinations is not satisfactory [10]. In contrast to the above anatomical imaging modalities, technetium-99 m methoxyisobutylisonitrile (^99m^Tc-MIBI) scintigraphy is a functional exploration and is regarded as the main preoperative localization method for patients with primary hyperparathyroidism (PHPT) or SHPT [11]. When combined with single-photon emission computed tomography/computed tomography (SPECT/CT), functional and anatomical, the sensitivity of scintigraphy is increased. Multiple studies have reported that dual-phase ^99m^Tc-MIBI scintigraphy is superior to US, especially when combined with SPECT/CT, in patients with HPT [12–14], but these studies did not distinguish SHPT from PHPT for statistical analysis. For PHPT, several investigations have reported that ^99m^Tc-MIBI SPECT/CT is superior to dual-phase ^99m^Tc-MIBI scintigraphy [11, 15, 16].

PHPT is mostly caused by parathyroid adenoma, which can usually be identified; however, SHPT usually involves more than one lesion, and it is difficult to identify all of the abnormal parathyroid glands because parathyroid hyperplasia is an asynchronous and asymmetrical process [17–19]. For SHPT, Jae Bok et al. [17] reported that US (91.5%) had a higher sensitivity than dual-phase ^99m^Tc-MIBI scintigraphy (56.1%), while Vulpio et al. [20] reported a slightly higher sensitivity for dual-phase ^99m^Tc-MIBI scintigraphy (62%) than US (55%). When combined with SPECT/CT, ^99m^Tc-MIBI SPECT/CT had a higher sensitivity than US [21, 22] and dual-phase ^99m^Tc-MIBI scintigraphy [6]. However, in all of these studies, only a single set of SPECT/CT images was acquired, either early or delayed. One prior investigation directly compared early and delayed ^99m^Tc-MIBI SPECT/CT and indicated that both early and delayed imaging should be performed [23]. The above studies reveal the value of SPECT/CT in patients with SHPT, but which phase of SPECT/CT is better in SHPT remains to be elucidated.

The purpose of this investigation was to directly compare the diagnostic performance of US, dual-phase ^99m^Tc-MIBI scintigraphy, early and delayed ^99m^Tc-MIBI SPECT/CT to obtain an exact preoperative map for the localization of abnormal parathyroid glands in SHPT and to determine whether dual-phase SPECT/CT is essential.

## Methods

### Clinical materials

From May 2017 to November 2019, a total of 60 CKD patients who were undergoing dialysis and parathyroidectomy for SHPT at the Sixth Affiliated Hospital of Sun Yat-sen University were included in this study. All patients underwent US, dual-phase ^99m^Tc-MIBI scintigraphy, early and delayed ^99m^Tc-MIBI SPECT/CT, and SHPT was confirmed by pathology. Patient demographics (sex, age, dialysis vintage), imaging data, laboratory values, and operative and pathological results were collected. Laboratory values included serum calcium, phosphorus, creatinine, alkaline phosphatase (ALP), and PTH after the last dialysis before surgery and postoperative PTH within the first week.

### Imaging methods

All patients with SHPT received an intravenous injection of 555 MBq ^99m^Tc-MIBI. Dual-phase ^99m^Tc-MIBI scintigraphy was obtained at 15 min and 120 min after injection. SPECT/CT integrated imaging was performed immediately after early and delayed ^99m^Tc-MIBI scintigraphy. Images were acquired using a Symbia Intevo 6 system (Siemens Healthcare) at an energy peak of 140 keV, window width of 20%, matrix of 128 × 128, magnification of 1-fold, and 600 k counts per frame with low-energy, high-resolution collimation. The CT scanning parameters were set at a field of view (FOV) of 40 cm, tube current of 200 mA, tube voltage of 130 kV, slice thickness of 2.5 mm, reconstruction matrix of 128 × 128, and reconstruction thickness of 2.5 mm. Imaging data were reconstructed using flash 3D. US was performed using a LOGIQ E9 system (GE Healthcare, USA) equipped with a 9 L linear probe (8.4–9 MHz) and ML6–15 probe (10–15 MHz). The images were analysed by professional doctor who were blinded to the laboratory, surgical, and pathological results.

### Image analysis

#### Dual-phase ^99m^Tc-MIBI scintigraphy

The image was considered positive on visual analysis when it met one of the following criteria: (1) abnormal ^99m^Tc-MIBI uptake was observed on both early and delayed imaging; (2) abnormal ^99m^Tc-MIBI uptake was observed on either early or delayed imaging. The image was considered negative when abnormal ^99m^Tc-MIBI uptake was observed on neither early nor delayed imaging [13].

#### Early and delayed ^99m^Tc-MIBI SPECT/CT

SHPT was positively diagnosed on early or delayed ^99m^Tc-MIBI SPECT/CT if CT indicated a parenchymal space-occupying lesion in the parathyroid region, while SPECT showed abnormal ^99m^Tc-MIBI accumulation compared to neck muscles and blood vessels. The result was considered negative if abnormal ^99m^Tc-MIBI uptake was observed on neither early nor delayed SPECT when CT indicated a parenchymal space-occupying lesion in the parathyroid region and if abnormal ^99m^Tc-MIBI uptake was observed on both early and delayed imaging but no parenchymal space-occupying lesion in the parathyroid region was observed on CT [21].

#### Us

A typical US image demonstrated an oval or asymmetrical hypoechoic mass at the upper and lower pole of the posterior thyroid with variable dimensions and separated by the thyroid gland. The lesion may rarely present with cystic degeneration. Colour Doppler examination showed a parathyroid vascular pedicle and a vascular arch located at the periphery of the gland [24].

#### Definition of ectopic parathyroid glands

Parathyroid glands were considered eutopic when the lower glands were related to the lower pole of the thyroid gland and when the upper glands were found near the upper pole of the thyroid. Hyperplastic parathyroid glands located inside the superior mediastinum region and the thyroid gland were regarded as ectopic parathyroid glands [6, 25].

#### Parathyroidectomy and final diagnosis

Parathyroidectomy was performed in severe SHPT patients who failed to respond to medical therapy. Our operative indications included persistent serum PTH > 800 pg/mL nonresponsive to medical therapy and at least one enlarged parathyroid gland confirmed on imaging [26]. In our study, serum PTH levels less than 300 pg/mL detected in the first postoperative week served as indicators of successful parathyroidectomy [27]. All patients underwent surgery by the same surgical team. Subtotal or total parathyroidectomy with autotransplantation was performed. In subtotal parathyroidectomy, 3 glands and half of the fourth gland were removed, while half of the normal gland was left in situ. Parathyroid glands resected during surgery were examined by pathology for the final diagnosis. The US, dual-phase ^99m^Tc-MIBI scintigraphy, early and delayed ^99m^Tc-MIBI SPECT/CT findings for each gland were defined as true positive, false positive, true negative, or false negative on the basis of the pathology results. Comparisons of the sensitivity (number of true-positives divided by number of true-positives plus false-negatives), specificity (number of true-negatives divided by number of true-negatives plus false-positives) and accuracy (number of true-positives plus true-negatives divided by number of total lesions resected) between groups were made according to the parathyroid pathology results.

#### Statistical analysis

Metric data are expressed as the median (25–75th percentile). Categorical variables were analysed using the χ2 or McNemar’s test. Spearman correlation was used for statistical analysis. A *P* value less than 0.05 was considered to indicate statistical significance. Statistical analysis was performed using IBM SPSS version 20.0 statistical software.

## Results

In all 60 patients included in the study, the primary disease was stage 5 CKD. The clinical pathological characteristics of the patients with SHPT who underwent surgery are summarized in Table 1. There were 34 males and 26 females, with a median age of 48 years and a median dialysis vintage of 7 years. Fifty-one patients underwent regular hemodialysis, 5 underwent peritoneal dialysis, and 4 underwent both hemodialysis and peritoneal dialysis. All patients had significantly increased serum PTH preoperatively and decreased serum PTH postoperatively. A total of 23% (14/60), 95% (57/60) and 74% (40/54, 6 patients were not measured before surgery) of patients showed increased serum calcium, phosphorus and ALP levels, respectively. Moreover, 23 patients showed punctate and annular calcification in the hyperplastic parathyroid glands, and the correlations between calcification and laboratory parameters, such as serum PTH, calcium, phosphorus, ALP and creatinine, were analysed. Spearman correlation results showed a significant albeit weak association between calcification and both serum PTH (*r =* 0.398, *P =* 0.002) and serum ALP (*r =* 0.415, *P =* 0.002), while no significant correlation was observed between calcification and serum calcium, phosphorus, or creatinine. In addition, a significant albeit weak association was observed between serum PTH and ALP (*r =* 0.349, *P =* 0.011).

**Table 1:** Baseline patient characteristics

Patient characteristics	Total
	(*n* = 60)
Age (yr)	48 (38–57.8)
Male sex, n (%) ^a^	34 (56.7%)
Type of dialysis, n (%)	
HD	51 (85.0%)
PD	5 (8.3%)
HD and PD	4 (6.7%)
Years of dialysis (yr)	7 (4–8.8)
Pre-op PTH level (pg/mL)	1602 (1282.24–2154.52)
Post-op PTH level (pg/mL)	8.35 (4.07–34.24)
Pre-op Creatinine (μmol/L)	949.70 (774.72–1180.77)
Pre-op ALP (U/L)	328.85 (225.61–730.74)
Pre-op Ca level (mmol/L)	2.42 (2.28–2.59)
Pre-op P level (mmol/L)	2.52 (2.09–3.00)
Location	
Eutopic	209
Ectopic	20
Superior mediastinum	15
Intrathyroid	5

Data are shown as the median (25–75th percentile). ^a^Number (%); Pre-op: preoperative; Post-op: postoperative. Normal range for PTH (12–88 pg/mL), creatinine (44–133 μmol/L), ALP (0–240 U/L), Ca (2.08–2.80 mmol/L), and P (0.83–1.96 mmol/L). *HD* Hemodialysis; *PD* Peritoneal dialysis

**Fig. 1:**
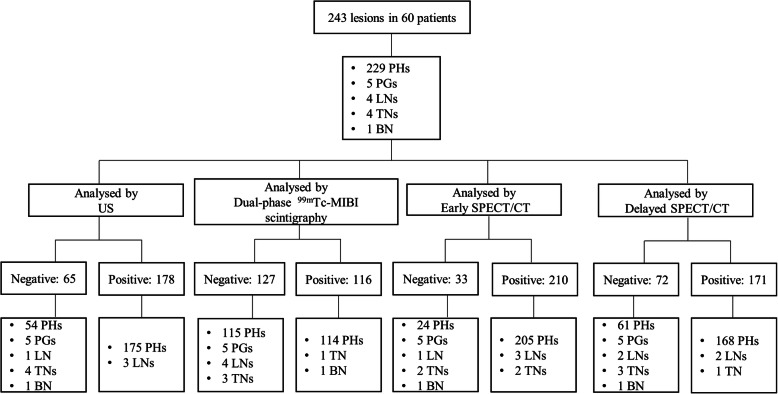
Flow chart of lesions analysed by US, dual-phase planar imaging, early SPECT/CT and delayed SPECT/CT. PH, parathyroid hyperplasia; PG, parathyroid gland; LN, lymph node; TN, thyroid nodule; BN, bone tissue

**Fig. 2:**
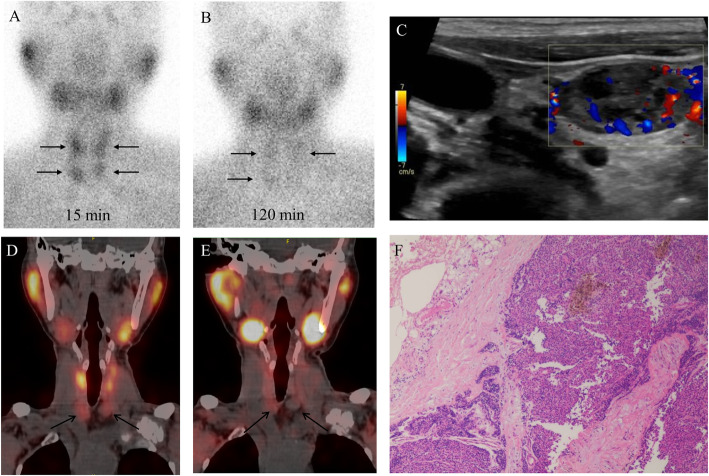
Representative images of a middle-aged patient with SHPT. Dual-phase ^99m^Tc-MIBI scintigraphy (A and B) showed 4 hyperplastic parathyroid glands. US (C) showed a typical enlarged parathyroid gland on the right. Early SPECT/CT (D) showed right inferior and left inferior hyperplastic parathyroid glands with ^99m^Tc-MIBI uptake. Delayed SPECT/CT (E) showed right inferior hyperplastic parathyroid gland with ^99m^Tc-MIBI uptake and left inferior hyperplastic parathyroid gland without ^99m^Tc-MIBI uptake. Pathological examination (F) confirmed nodular parathyroid hyperplasia

In all, 59 of the 60 patients underwent total parathyroidectomy with autotransplantation, and one patient underwent subtotal parathyroidectomy. Meanwhile, 8 patients underwent partial thyroidectomy, and 1 patient with papillary thyroid carcinoma underwent total thyroidectomy. As shown in Fig. 1, a total of 243 lesions were resected from the 60 patients; 229 lesions were pathologically confirmed to be parathyroid hyperplasia, 5 were normal parathyroid glands, 4 were lymph nodes, 4 were thyroid nodules and 1 was bone tissue. Of the 229 confirmed lesions, 209 were in typical sites, 15 were in the upper mediastinum, and 5 were in the thyroid gland. Among the 60 patients, four lesions were identified in 53 patients (88.33%), two lesions were identified in 4 patients (6.67%), and the other three patients had 1, 3, and 5 lesions, respectively. US showed 178 positive lesions in 60 patients, and 175 lesions were confirmed to be true positive. The three false-positive lesions were confirmed to be lymph nodes. Dual-phase planar imaging detected 116 positive lesions, and there were 2 false-positive lesions. One was confirmed to be thyroid nodule, and the other was due to the focal ^99m^Tc-MIBI uptake of the manubrium sterni without destruction of bone observed on SPECT/CT. Early ^99m^Tc-MIBI SPECT/CT showed 210 positive lesions, and 205 lesions were confirmed to be true-positive lesions. For the 5 false-positive lesions, three lesions were confirmed to be lymph nodes, and two lesions were thyroid nodules. Delayed ^99m^Tc-MIBI SPECT/CT showed 171 positive lesions, including 168 true-positive and 3 false-positive lesions, of which two lesions were confirmed to be lymph nodes and the other was a thyroid nodule. Representative images are shown in Fig. 2.

Analysed by case number, the sensitivity of US, dual-phase ^99m^Tc-MIBI scintigraphy, early and delayed ^99m^Tc-MIBI SPECT/CT was 93.33% (56/60), 76.67% (46/60), 98.33% (59/60), and 98.33% (59/60), respectively. Analysed by lesion number, early ^99m^Tc-MIBI SPECT/CT showed the highest sensitivity and accuracy (89.5 and 88.0%), while dual-phase ^99m^Tc-MIBI scintigraphy showed the lowest (49.8 and 51.9%). US, early and delayed ^99m^Tc-MIBI SPECT/CT showed significantly higher sensitivity and accuracy than dual-phase ^99m^Tc-MIBI scintigraphy (*P <* 0.001). Furthermore, early ^99m^Tc-MIBI SPECT/CT showed significantly higher sensitivity (*P <* 0.001) and accuracy (*P =* 0.001 and *P <* 0.001) than US and delayed ^99m^Tc-MIBI SPECT/CT (Table 2). There was no significant difference in sensitivity or accuracy between US and delayed ^99m^Tc-MIBI SPECT/CT. The specificity of US, dual-phase ^99m^Tc-MIBI scintigraphy, early and delayed ^99m^Tc-MIBI SPECT/CT was 78.6, 85.7, 64.3 and 78.6%, respectively.

**Table 2:** Preoperative imaging and postoperative parathyroid pathology results

	US	Dual-phase ^99m^Tc-MIBI scintigraphy	Early SPECT/CT	Delayed SPECT/CT
TP	175	114	205	168
FP	3	2	5	3
FN	54	115	24	61
TN	11	12	9	11
Sensitivity (%)	76.40%	49.80%	89.50%	73.40%
Specificity (%)	78.60%	85.70%	64.30%	78.60%
Accuracy (%)	76.50%	51.90%	88.00%	73.70%

*TP* True positive; *FP* False positive; *FN* False negative; *TN* True negative

Then, the sensitivity of preoperative eutopic and ectopic parathyroid imaging was compared (Table 3). Twenty ectopic parathyroid glands (20/229, 8.73%) were detected in 16 patients (16/60, 26.67%). The sensitivity of parathyroid imaging for eutopic lesions (US, 78.5%; dual-phase ^99m^Tc-MIBI scintigraphy, 49.8%; early ^99m^Tc-MIBI SPECT/CT, 89.5%; and delayed ^99m^Tc-MIBI SPECT/CT, 73.2%) was similar to the sensitivity of imaging for all lesions (US, 76.4%; dual-phase ^99m^Tc-MIBI scintigraphy, 49.8%; early ^99m^Tc-MIBI SPECT/CT, 89.5%; and delayed ^99m^Tc-MIBI SPECT/CT, 73.4%). However, for ectopic parathyroid lesions, the sensitivity of early ^99m^Tc-MIBI SPECT/CT was significantly higher than that of US and dual-phase ^99m^Tc-MIBI scintigraphy (*P <* 0.05). Delayed ^99m^Tc-MIBI SPECT/CT was not significantly superior to US or dual-phase ^99m^Tc-MIBI scintigraphy in terms of sensitivity.

**Table 3:** Comparison of eutopic and ectopic parathyroid glands among the four techniques

		US	Dual-phase ^99m^Tc-MIBI scintigraphy	Early SPECT/CT	Delayed SPECT/CT
Eutopic parathyroid glands	TP	164	104	187	153
	FN	45	105	22	56
	Sensitivity (%)	78.50%	49.80%	89.50%	73.20%
Ectopic parathyroid glands	TP	11	10	18	15
	FN	9	10	2	5
	Sensitivity (%)	55.00%	50.00%	90.00%	75.00%

*TP* True positive; *FN* False negative

## Discussion

SHPT is a common complication of CKD and is followed by disorders of calcium and phosphorus metabolism, abnormal PTH secretion, and parathyroid hyperplasia. Serum PTH plays a critical role in the maintenance of calcium and phosphate levels. Unlike PHPT, which is independent of PTH secretion by the parathyroid tissue, in SHPT, compensatory PTH secretion occurs due to hypocalcaemia [22]. Therefore, in patients with SHPT, the serum calcium level can be increased or remain normal. In our study, all patients had increased serum PTH, but only 23.33% of patients showed increased serum calcium. We found that 23 patients showed punctate and annular calcification in the hyperplastic parathyroid glands, and annular calcification might be a special sign of SHPT [28–30]. Soft tissue and vascular calcification is commonly present in end-stage renal disease, secondary to disturbances in the calcium and phosphate balance and HPT, which may explain why the parathyroid glands were more prone to calcification in patients with SHPT [31, 32]. A previous study showed an association between serum ALP and vascular calcification via modulation of the pyrophosphate pathway [33, 34]. Serum PTH can increase bone metabolic conversion to elevate the serum ALP level [22]. Therefore, as we reported, calcification of the parathyroid glands might be correlated with serum PTH and ALP.

For severe SHPT, parathyroidectomy remains the best treatment option when drug treatment fails. However, surgical results among SHPT patients are less satisfactory compared with PHPT due to the incomplete intraoperative identification of all parathyroid glands [23]. Therefore, preoperative imaging and localization are critical to a successful operation. US is the most commonly used imaging modality due to its advantages of low cost and simple manipulation, but it is limited in the detection of ectopic parathyroid glands and is dependent on the examiner’s experience [24]. CT may be necessary to locate parathyroid glands precisely, especially for the ectopic glands in the mediastinum [7]. MRI has the advantage of no radiation and a slightly greater sensitivity than CT, and it is especially useful in detecting mediastinal parathyroid glands [24]. Different from the above anatomical imaging modalities, ^99m^Tc-MIBI imaging is a functional exploration with the advantage of detecting ectopic glands and is based on the different washout rates between the thyroid tissue and hyperplastic parathyroid tissue [35]. Dual-phase ^99m^Tc-MIBI scintigraphy has high sensitivity and specificity but also has multiple limitations, such as the lack of a precise anatomical location of the lesion [36], which increased the difficulty of distinguishing hyperplastic parathyroid lesions from thyroid lesions. ^99m^Tc-MIBI SPECT/CT is significantly superior to planar imaging in the detection of parathyroid abnormalities because it provides more precise anatomical localization, particularly for ectopic lesions, as well as the identification of supernumerary glands and parathyroid glands with the lowest ^99m^Tc-MIBI uptake [11, 37].

In our study, we directly compared US, dual-phase ^99m^Tc-MIBI scintigraphy, early and delayed ^99m^Tc-MIBI SPECT/CT in SHPT patients. The overall sensitivity and accuracy of early SPECT/CT were higher than those of the other techniques and slightly higher than those in previous studies [23, 24]. For detecting ectopic parathyroid glands, the sensitivity of early ^99m^Tc-MIBI SPECT/CT was significantly higher than that of US and dual-phase ^99m^Tc-MIBI scintigraphy, and the percentage was similar to that reported in a previous study (90.5%) [6]. Our results show that early ^99m^Tc-MIBI SPECT/CT was superior to the other modalities in detecting parathyroid lesions in SHPT patients. Hybrid SPECT/CT can provide not only functional information acquired through SPECT but also an accurate anatomical depiction of the parathyroid gland location and size and adjacent structures through CT, especially in cases of ectopic and supernumerary parathyroid glands. With delayed ^99m^Tc-MIBI SPECT/CT, two investigational groups reported a high sensitivity of 59.3% [22] and 85% [21], and in our study, the sensitivity was 73.4%. In our investigation, we found that the ^99m^Tc-MIBI uptake of some hyperplastic parathyroid glands on early ^99m^Tc-MIBI SPECT/CT was slightly higher than the background uptake but lower than the thyroid uptake and that further clearance in delayed SPECT/CT caused false-negative results on delayed SPECT/CT. Schachter et al. reported that delayed ^99m^Tc-MIBI SPECT/CT may be nondiagnostic when similar washout rates are found in thyroid and parathyroid tissue [38]. Therefore, dual-phase ^99m^Tc-MIBI scintigraphy in combination with early ^99m^Tc-MIBI SPECT/CT may be considered as a valuable preoperative evaluation method. Meanwhile, dual-phase ^99m^Tc-MIBI scintigraphy cannot be replaced by early ^99m^Tc-MIBI SPECT/CT because the former can roughly show the location of parathyroid hyperplasia especially ectopic parathyroid hyperplasia, and help to determine the SPECT/CT scan range.

Most patients in our study had four proven lesions, which reminds us to identify as many as four parathyroid lesions as possible when diagnosing SHPT. As we found that all of the calcified parathyroid glands (45 lesions) were confirmed to be parathyroid hyperplasia by pathology, if calcified nodules are observed in the parathyroid region, parathyroid hyperplasia should be suspected. However, large-sample, multicentre studies are still needed to confirm these findings.

The main reason for the low sensitivity of US is believed to be the frequent misdiagnosis of inferior parathyroid lesions, especially mediastinal ectopic parathyroid glands. The examiner’s experience in the accurate determination of lesions is also a critical factor that cannot be ignored. It was reported that the sensitivity of US for diagnosing SHPT ranged from 46.24 to 91.5% [17, 21, 22], which is similar to our result (75.65%).

Our observations show that the sensitivity of dual-phase ^99m^Tc-MIBI scintigraphy was the lowest of the four modalities. The reasons are as follows. First, although dual-phase ^99m^Tc-MIBI scintigraphy can effectively detect ectopic parathyroid glands, only a general increase in radioactivity can be observed, and the number of lesions cannot be clearly distinguished when multiple parathyroid gland lesions are adjacent to each other. Second, some hyperplastic parathyroid glands were positive for p-glycoprotein, and ^99m^Tc-MIBI was quickly eliminated from the parathyroid glands, leading to negative uptake images on scintigraphy [39]. Third, the findings on dual-phase ^99m^Tc-MIBI scintigraphy are related to the size and weight of the parathyroid glands, and smaller parathyroid gland lesions can be easily missed [24, 40]. Fourth, lesions located behind the thyroid gland and with similar ^99m^Tc-MIBI uptake are difficult to detect on scintigraphy but can be identified on SPECT/CT. In a meta-analysis, the pooled sensitivity of dual-phase ^99m^Tc-MIBI scintigraphy in SHPT was 58% [41], which is slightly higher than our result.

David Taieb et al. [40] reported that the most common causes of false-positive results on parathyroid scintigraphy were the presence of thyroid nodules, thymoma, metastatic and inflammatory lymph nodes, while skeletal brown tumours may also be a rare cause of false-positive lesions. However, in our study, multiple false-positive lesions found on US, early and delayed ^99m^Tc-MIBI SPECT/CT were mostly confirmed to be lymph nodes, followed by thyroid nodules. The most likely explanation for this difference is that we identified fewer false-positive lesions, and all our patients were SHPT patients and not PHPT patients. One false-positive lesion found on dual-phase ^99m^Tc-MIBI scintigraphy was attributed to the focal ^99m^Tc-MIBI uptake of the manubrium sterni. A previous study suggested that the ^99m^Tc-MIBI uptake of bone might reflect the presence of active metabolic bone disease but did not reflect changes that occurred in the microstructure of bone [42]. However, this remains to be evaluated further. In addition, the specificity of early ^99m^Tc-MIBI SPECT/CT in our study is lower than that in a previous study (75%) [6], probably due to the small number of true-negative cases.

Our study is limited by its retrospective design and relatively small cohort of patients. Therefore, future prospective randomized studies of preoperative imaging modalities are needed for more accurate and objective investigations.

## Conclusion

Our study demonstrates that the ability of early ^99m^Tc-MIBI SPECT/CT to detect parathyroid lesions in SHPT patients is superior to that of US, dual-phase ^99m^Tc-MIBI scintigraphy and delayed ^99m^Tc-MIBI SPECT/CT. In cases of ectopic hyperplastic parathyroid glands, the sensitivity of early ^99m^Tc-MIBI SPECT/CT (90%) was significantly higher than that of US (55%) and dual-phase ^99m^Tc-MIBI scintigraphy (50%). These findings suggest that dual phase ^99m^Tc-MIBI scintigraphy, with early ^99m^Tc-MIBI SPECT/CT may be considered as part of the preoperative evaluation of patients with SHPT. The results of our small-sample study suggest that parathyroid gland calcification may be related to the levels of PTH and ALP and could help provide evidence for the identification of SHPT.

## Data Availability

Data and materials during the current study are available from the corresponding author upon reasonable request.

## Abbreviations

^99m^Tc-MIBI: Technetium-99 m methoxyisobutylisonitrile
SHPT: Secondary hyperparathyroidism
PHPT: Primary hyperparathyroidism
ALP: Alkaline phosphatase
PTH: Parathyroid hormone
US: Ultrasonography
CT: Computed tomography
MRI: Magnetic resonance imaging
SPECT/CT: Single-photon emission computed tomography/computed tomography
CKD: Chronic kidney disease
